# Three-dimensional femur morphology analysis for the optimal location of subtrochanteric osteotomy with an implanted Wagner cone stem in total hip arthroplasty for Crowe type IV developmental dysplasia of the hip

**DOI:** 10.1186/s13018-023-03901-7

**Published:** 2023-06-05

**Authors:** Kun Tao, Shi-Cheng Wang, Xiao-Ying Ma, Long Shao, Zheng-Lin Di, Zhe-Yu Huang

**Affiliations:** 1 The Department of Orthopedics Surgery, Ningbo No. 6 Hospital, 1059# ZhongShan East Road, Ningbo, 315040 China; 2 Beijing Naton Medical Technology Holdings Co., Ltd., Beijing, 100094 China

**Keywords:** Developmental dysplasia of the hip, Subtrochanteric osteotomy, Non-union, Three-dimensional femur morphology, Optimal location

## Abstract

**Background:**

This study aimed to accurately evaluate the matching of proximal and distal femoral segments and fitting of the femur–femoral stem in patients with Crowe type IV developmental dysplasia of the hip (DDH) who have undergone subtrochanteric osteotomy at different locations with an implanted Wagner cone stem to improve the rate of the bone union at the osteotomy site.

**Methods:**

Three-dimensional femur morphology of 40 patients with Crowe type IV DDH was evaluated at each cross-section to determine the femoral cortical bone area. This study focused on five osteotomy lengths (2.5, 3, 3.5, 4, and 4.5 cm). The overlapped area between the proximal and distal cortical bone segments was defined as the contact area (*S*, mm^2^), and the contact area to distal cortical bone area ratio was defined as the coincidence rate (*R*). Three indicators were used to evaluate the matching and fitting of the osteotomy sites with the implanted Wagner cone stems: (1) higher *S* and *R* between the proximal and distal segments; (2) the effective fixation length of the femoral stem at the distal segments being at least 1.5 cm; and (3) osteotomy did not involve the isthmus.

**Results:**

In all groups, *S* significantly decreased in the two proximal levels above the 0.5 cm level below the lesser trochanter (LT) compared with those below this level. In comparison, at osteotomy lengths from 2.5 to 4 cm, *R* significantly decreased in the three proximal levels. The optimal osteotomy levels ranged from 1.5 and 2.5 cm below the LT for an appropriately sized stem.

**Conclusions:**

Subtrochanteric osteotomy at the optimal level not only ensures fitting of the femur–femoral stem but also meets the requirements of a higher *S* and *R* to ensure adequate reduction and stabilization at the osteotomy site, which may contribute to the bone union. Although the optimal osteotomy level varies with the size of the femoral stem and the length of the subtrochanteric osteotomy, the optimal osteotomy levels for an appropriately sized Wagner cone femoral stem implantation range from 1.5 to 2.5 cm below the LT.

**Supplementary Information:**

The online version contains supplementary material available at 10.1186/s13018-023-03901-7.

## Background

Crowe type IV developmental dysplasia of the hip (DDH) is characterized by (1) a triangular-shaped and shallow acetabulum, (2) narrower measures of the femoral canal, (3) excessive anteversion of the femur and valgus neck–shaft angle, (4) posterior location of the greater trochanter, (5) soft tissue contractures, (6) lower limb length discrepancy, and (7) hip abductor mechanism insufficiency [1, 2]. Such problems cause technical difficulties in total hip arthroplasty (THA) during length inequality correction and placement of the acetabular component in the true acetabulum [3]. When restoring the anatomical center of hip rotation, the leg may be lengthened by > 4 cm [4], which increases the risk of direct or indirect neurologic injury [5]. Consequently, subtrochanteric femoral shortening osteotomy combined with a modular hip system or straight cone femoral stem is usually recommended for the treatment of Crowe type IV DDH because it facilitates the pulling down of the femur. This corrects the rotational abnormalities, preserves the proximal femoral metaphysis, and reduces the risk of nerve injury [6, 7].

Subtrochanteric transverse osteotomy is commonly performed because it is technically simple, can be performed repeatedly, and causes minimal damage to the periosteum at the osteotomy site [8, 9]. However, a limited bone contact area and low coincidence rate between the proximal and distal fragments are the major disadvantages of transverse osteotomy, which may interfere with bone healing [10–12]. A study has shown that different levels and lengths of femoral osteotomy produce different bone contact areas and coincidence rates [12]. However, their findings were based on X-ray data, which are subject to error and do not take into account the implantation of femoral stems, such as the Wagner cone femoral stem (Zimmer Biomet, Warsaw, IN, USA), which is usually recommended for Crowe type IV DDH [7, 13]. Previous studies have shown that there is no standard osteotomy location, and the location is determined by the experience of clinicians [6, 14–16]. Therefore, this study aimed to evaluate the matching and fitting of the osteotomy site in patients with Crowe type IV DDH who have undergone subtrochanteric osteotomy using Wagner cone femoral stems of different sizes under different osteotomy parameters (levels and lengths) and to analyze the optimal osteotomy location to ensure sufficient reduction and stabilization at the osteotomy site.

## Methods

### Participants

We searched our Medical Image Database using our hospital’s picture archiving and communication system. Next, the CT images of 40 adult patients (40 hips) diagnosed with Crowe type IV DDH according to the Crowe classification method were retrospectively reviewed [17] from January 2016 to May 2022. There were 32 females and eight males, with an average age of 57.5 ± 10.3 (32–73) years. The inclusion criteria were: (1) adult patients with unilateral Crowe type IV DDH and (2) patients whose CT images were available, and (3) CT images with the scanning range covering the proximal femur to the femoral isthmus. The exclusion criteria included: (1) previous hip or pelvic surgery, (2) residual DDH due to infection or trauma and flexion contracture of the hip, and (3) history of cerebral palsy, poliomyelitis, and other nervous system diseases. The study protocol was approved by our facility’s institutional review board, and written informed consent was obtained from all patients.

### Reconstruction of the 3D femur morphology model using CT images

**Fig. 1:**
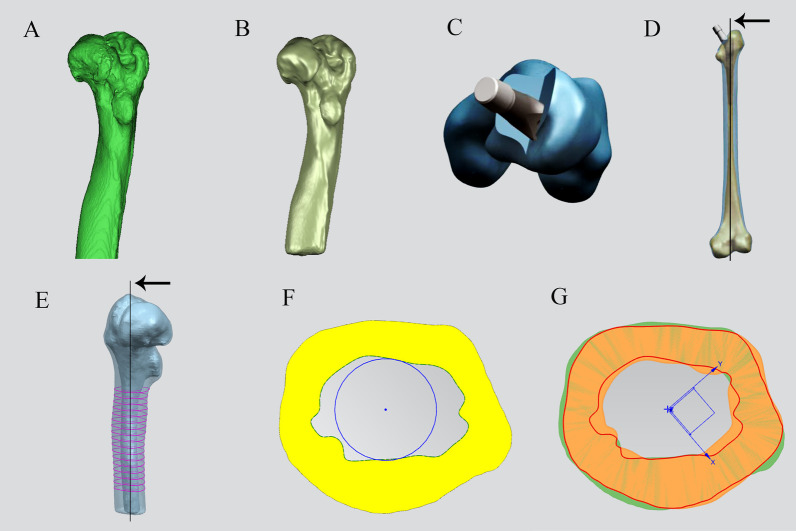
**A** Three-dimensional reconstruction femoral model generated using Mimics 21.0 and **B** smoothed using Geomagic Wrap. **C** The Wagner cone stem is implanted into the femoral medullary cavity with a 20° anteversion angle. **D** The implant axis of the femur is established after the Wagner cone stem is implanted. **E** Seventeen selected levels from 0 to 8 cm below the LT at 0.5 cm intervals. **F** The center point circle method for the 17 selected levels. **G** The central points of the medullary cavity are overlapped in pairs according to the osteotomy length. A pruning tool is used to obtain the corresponding overlap area of cortical bone. The black arrow indicates the implant axis of the femur

The hips were scanned from the pelvis to the femoral isthmus with all participants in a supine position, with the lower limbs in the same width as the pelvis using a multislice CT scanner (Philips Brilliance 64 CT; Philips Medical Systems, Eindhoven, Netherlands), with the following parameters: scan voltage, 120 kV; scan current, 60 mA; matrix, 512 × 512; and slice thickness, 0.625 mm. All data were obtained in a DICOM file format and imported into Mimics 21.0 (Materialize, Leuven, Belgium) to generate a 3D reconstruction model of the femur. In Mimics, the femur was isolated from surrounding bone and soft tissues; then, the medial and lateral boundaries of each layer of femoral cortical bone were manually examined and modified until a 3D femoral model was automatically reconstructed based on the default optimal settings. The femoral model was transferred in a stereolithography format to Geomagic Wrap (3D Systems Inc., Rock Hill, SC, USA) for analysis. The surface of the femoral model was smoothed through a series of procedures called “Mesh doctor,” → “Remove spikes,” → “Fill holes,” etc. A femoral model was created for each participant (Fig. 1).

### Establishment of an implant axis of the femur

First, all femoral models were transferred in an Initial Graphics Exchange Specification format to Unigraphics NX version 1847 (Siemens PLM Software, Co, Ltd, Plano, TX). Next, femoral neck osteotomy was simulated at the proximal section of the lesser trochanter (LT). Then an appropriate femoral stem was implanted into the femoral medullary cavity with a 20° anteversion angle. Finally, the stem axis was determined as the implant axis of the femur (Fig. 1).

### Evaluation of bone matching at the osteotomy site

Bone matching at the osteotomy site was represented by three parameters: the cortical bone area at each level, contact area, and coincidence rate. First, 17 levels perpendicular to the implant axis of the femur were selected from 0 to 8 cm below the LT at 0.5 cm intervals. Second, the cortical bone area at each level was calculated using a measuring tool. Third, the central points, i.e., the intersection point of the implant axis and the cross-section, were overlapped in pairs according to the osteotomy length. A pruning tool was used to obtain the corresponding overlap area of cortical bone (Fig. 1). Clinically, the most common osteotomy length was 2.5–4.5 cm [7, 18, 19]; therefore, this study mainly focused on bone matching at the osteotomy site at five osteotomy lengths (2.5, 3, 3.5, 4, and 4.5 cm), corresponding to five groups (i.e., 2.5L, 3L, 3.5L, 4L, 4.5L). Finally, the overlap area, which was the contact area (*S*, mm^2^), was measured for all the groups; the contact area to distal cortical bone area ratio was also identified as the coincidence rate (*R*).

### Evaluation of the femur–femoral stem fitting

Three parameters represented the femur–femoral stem fitting: (1) the shortest diameter of the medullary cavity (SDMC), (2) the diameter of the Wagner cone femoral stem, and (3) the optimal osteotomy location for the effective fixation length (EFL) of the femoral stem at the distal segments being ≥ 1.5 cm.

**Fig. 2:**
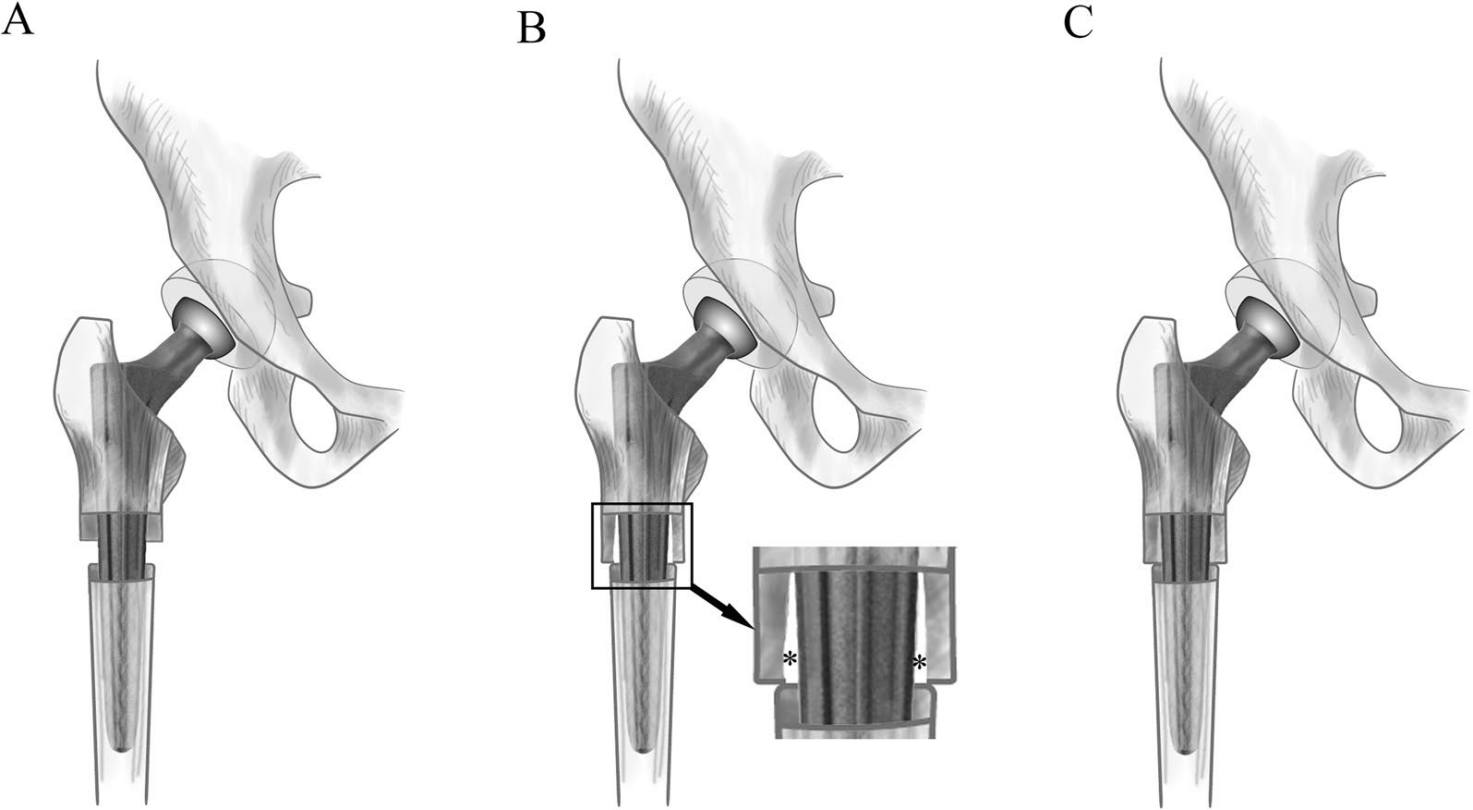
**A** Separation of the proximal and distal segments for smaller SDMCs (i.e., the SDMC is smaller than the femoral stem diameter by ≥ 2 mm). **B** Poor femoral stem fixation for larger SDMCs. **C** Adequate matching of the proximal and distal segments with an appropriately sized Wagner cone femoral stem. *Untouched area of the femur and stem

**Fig. 3:**
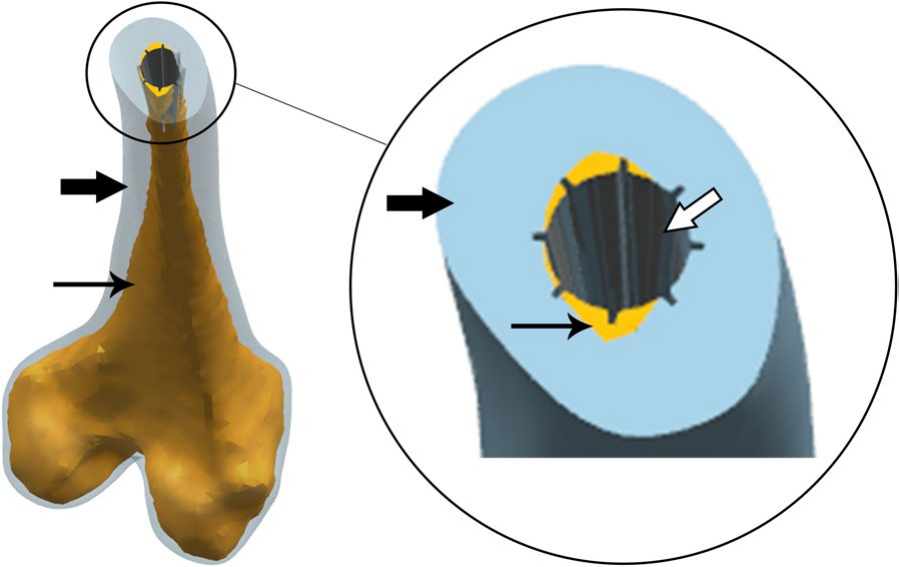
Effective fixation for the femur–femoral stem fitting. The thick black arrows indicate cortical bone, the thin black arrows indicate the Medullary cavity, and the thick white arrow indicates the Wagner cone stem

The diameter of the largest inscribed circle for each level of the medullary cavity was also measured when measuring the cortical bone area at each level (Fig. 1F). The diameters of the inscribed circles were defined as the SDMCs. The diameter of the Wagner cone stem for each level was also measured, and a total of 12 stem diameters (13–24 mm) were analyzed. The stem diameter was measured at 80.5 mm and 90 mm from the stem tips for sizes 13 and 14–24, respectively, and corresponded to the outer rib diameter. The rib height was 1, 1.5, 2, and 2.5 mm for sizes 13–15, 16–18, 19–22, and 23 and 24, respectively. With a cone angle of 5°, the distal stem diameter varied from 6.4 mm (size 13) to 17.4 mm (size 24) [20]. Therefore, the stem’s proximal to distal section diameter could be accurately calculated. Femoral neck osteotomy was set at the proximal section of the LT; the final position of the femoral stem in the femur was with the proximal–medial end of the stem being at the same level as the proximal end of the LT. The LT’s height (25.9 mm) was measured to further study the femur–femoral stem matching in the area below the LT (Additional file 1). For convenience, the LT height was set at 25 mm. Since the minimum rib height was 1 mm for Wagner cone femoral stems of sizes 13–15, the SDMC should be slightly less than (within 2 mm) or equal to the diameter of the femoral stem section at the same level. Otherwise, separation or periprosthetic fracture of the proximal and distal segments for smaller SDMCs (i.e., the SDMC is smaller than the femoral stem diameter by ≥ 2 mm) or poor fixation of femoral stems for larger SDMCs may occur (Fig. 2). This form of fixation in which the prosthesis was in full contact with cortical bone was called effective fixation (Fig. 3).

The stem length is vital to achieving stability; the femoral stem’s EFL at the distal segments mainly provides stability at the osteotomy site. Ozan et al. [21] recommended that the stem be bridged to the osteotomy site by at least 4–5 cm, which was not the EFL. Russell et al. [22] suggested that a minimum EFL of 1.5–2.5 cm is sufficient to obtain adequate initial fixation stability with a tapered stem design. Therefore, the minimum EFL was set at 1.5 cm.

Different osteotomy levels, osteotomy lengths, and stem sizes will result in different matching conditions between the femur and femoral stem. The SDMC was compared with the diameter of the Wagner cone femoral stem in each matching condition from the distal osteotomy level to a level 1.5 cm below the distal osteotomy level to determine the optimal osteotomy location for the femoral stem EFL at the distal segments ≥ 1.5 cm.

### Evaluation of femoral isthmus involvement during subtrochanteric osteotomy

Femoral isthmus integrity is crucial to the stability of the femoral prosthesis [23]. According to a study by Su et al. [24], a level 8 cm below the LT always reaches the femoral isthmus. To preserve the integrity of the femoral isthmus during subtrochanteric osteotomy, the level of distal osteotomy was not > 8 cm below the LT to obviate femoral isthmus involvement.

### Statistical analysis

The whole measurement process was repeated independently by two operators. Intraclass correlation coefficients were 0.879, 0.902, and 0.895 for *S*, *R*, and SDMC, respectively, suggesting adequate reliability across all measures [25]. Quantitative data are presented as means ± standard deviations and categorical variables as percentages. Statistical analyses were performed using SPSS v24.0 (IBM Inc., Armonk, NY, USA). For each group, intra-group comparisons of *S* and *R* values were performed separately using one-way analysis of variance (ANOVA) and the Student–Newman–Keuls test (*q* test). Statistical significance was set at *p* < 0.05.

## Results

### Bone matching at the osteotomy site

#### Cortical bone area at each level

The average cortical bone area at each level from the proximal to the distal femur is shown in Table 1. The maximum average area of cortical bone was 325 mm^2^, which was located just below the LT, whereas the smallest area (315 mm^2^) was located 1.5 cm below the LT. There was no statistical difference in cortical bone areas at each level.

**Table 1:** Bone Matching at Osteotomy Site

Level (cm)	Area of cortical bone (mm^2^)	Contact Area (mm^2^)					Coincidence rate				
		Length of Osteotomy					Length of Osteotomy				
		2.5 cm	3 cm	3.5 cm	4 cm	4.5 cm	2.5 cm	3 cm	3.5 cm	4 cm	4.5 cm
0	325 ± 57	202 ± 76	198 ± 77	195 ± 76	192 ± 74	190 ± 73	63%	61%	61%	60%	59%
0.5	316 ± 56	230 ± 69	226 ± 68	222 ± 67	218 ± 66	215 ± 65	72%	71%	69%	68%	67%
1	318 ± 62	253 ± 64	249 ± 62	245 ± 60	241 ± 60	239 ± 58	80%	78%	77%	75%	74%
1.5	315 ± 66	262 ± 60	258 ± 58	253 ± 58	250 ± 57	247 ± 57	82%	81%	79%	77%	77%
2	319 ± 60	270 ± 54	265 ± 53	263 ± 52	258 ± 53	254 ± 53	85%	83%	82%	81%	80%
2.5	317 ± 56	273 ± 51	271 ± 50	266 ± 50	263 ± 50	260 ± 52	86%	84%	84%	82%	81%
3	316 ± 56	280 ± 53	272 ± 51	268 ± 51	266 ± 54	254 ± 55	87%	85%	84%	83%	80%
3.5	316 ± 58	280 ± 54	275 ± 52	272 ± 54	260 ± 54	250 ± 56	88%	86%	85%	82%	79%
4	317 ± 55	282 ± 53	278 ± 54	269 ± 55	258 ± 55	NA	88%	86%	85%	81%	NA
4.5	318 ± 54	284 ± 55	274 ± 54	263 ± 55	NA	NA	88%	86%	83%	NA	NA
5	319 ± 57	279 ± 55	266 ± 55	NA	NA	NA	88%	84%	NA	NA	NA
5.5	322 ± 58	274 ± 55	NA	NA	NA	NA	86%	NA	NA	NA	NA
6	319 ± 58	NA	NA	NA	NA	NA	NA	NA	NA	NA	NA
6.5	320 ± 57	NA	NA	NA	NA	NA	NA	NA	NA	NA	NA
7	321 ± 59	NA	NA	NA	NA	NA	NA	NA	NA	NA	NA
7.5	318 ± 59	NA	NA	NA	NA	NA	NA	NA	NA	NA	NA
8	317 ± 59	NA	NA	NA	NA	NA	NA	NA	NA	NA	NA

*NA*—not available

#### Contact area (*S*)

In the 2.5L group, the minimum average S (202 mm^2^) was obtained at the level just below the LT; the average S value was 230 mm^2^ for the 0.5 cm level below the LT. S significantly decreased in the two proximal levels above the 0.5 cm level below the LT compared with those below this level (*p* < 0.0001). Similar results were also observed in the other groups, and the data are summarized in Table 1. The ANOVA and *q* test results for each group are shown in Additional files 2, 3, 4, 5, and 6.

#### Coincidence rate (*R*)

Statistically significant results were noted in the *R* values. In the 2.5L group, the minimum average *R* (63%) was observed at the level just below the LT; the average *R* values were 72% and 80% for the 0.5 and 1 cm levels below the LT, respectively. *R* significantly decreased in the three proximal levels above the 1 cm level below the LT compared with those below this level (*p* < 0.0001). Similar results were observed in the 3L, 3.5L, and 4L groups. In the 4.5L group, *R* significantly decreased in the two proximal levels above the 0.5 cm level below the LT compared with those below this level (*p* < 0.0001). These data are summarized in Table 1; the ANOVA and *q* test results for each group are shown in Additional files 2, 3, 4, 5, and 6.

### Femur–femoral stem fitting

#### SDMC

The maximum average SDMC was 14.2 mm, located just below the LT, whereas the smallest average SDMC (9.8 mm) was located 8 cm below the LT. The average SDMC values demonstrated a gradually decreasing trend from the proximal to the distal segments. Details of the measurements are summarized in Table 2.

**Table 2:** Femur–femoral stem matching

Level (cm)	Shortest diameter of the medullary cavity (mm)	Diameter of Wagner cone femoral stem^a^ (mm)		
		13#	14#	15#
0	14.2 ± 3.6	12.6	13.6	14.6
0.5	13.3 ± 3.4	12.1	13.1	14.1
1	12.5 ± 3.1	11.7	12.7	13.7
1.5	12.1 ± 3.0	11.3	12.3	13.3
2	11.5 ± 2.7	10.8	11.8	12.8
2.5	11.2 ± 2.6	10.4	11.4	12.4
3	11.1 ± 2.6	10.0	11.0	12.0
3.5	10.9 ± 2.7	9.5	10.5	11.5
4	10.7 ± 2.6	9.1	10.1	11.1
4.5	10.6 ± 2.6	8.7	9.7	10.7
5	10.5 ± 2.5	8.2	9.2	10.2
5.5	10.4 ± 2.4	7.8	8.8	9.8
6	10.3 ± 2.4	7.4	8.4	9.4
6.5	10.1 ± 2.3	6.9	7.9	8.9
7	10.0 ± 2.2	6.5	7.5	8.5
7.5	9.9 ± 2.3	NA	NA	NA
8	9.8 ± 2.2	NA	NA	NA

*NA*—not available

^a^The level 25 mm below the proximal–medial end of the Wagner cone femoral stem was the same level just below the lesser trochanter

#### Diameter of the Wagner cone femoral stems

The three smallest stems (13–15) were measured. At an LT height of 25 mm, the diameters measured at the level just below the LT were 12.6, 13.6, and 14.6 mm for sizes 13, 14, and 15, respectively. According to the parameters of the Wagner cone femoral stems, the distal stem diameter level was 7.1 cm below the LT. Therefore, the area 7 cm below the LT was set as the level of the distal stem diameter, and the following diameters were obtained: 6.5, 7.5, and 8.5 mm for sizes 13, 14, and 15, respectively. Details of the measurements are summarized in Table 2.

#### Optimal osteotomy location for the femoral stem EFL at distal segments of at least ≥ 1.5 cm

**Fig. 4:**
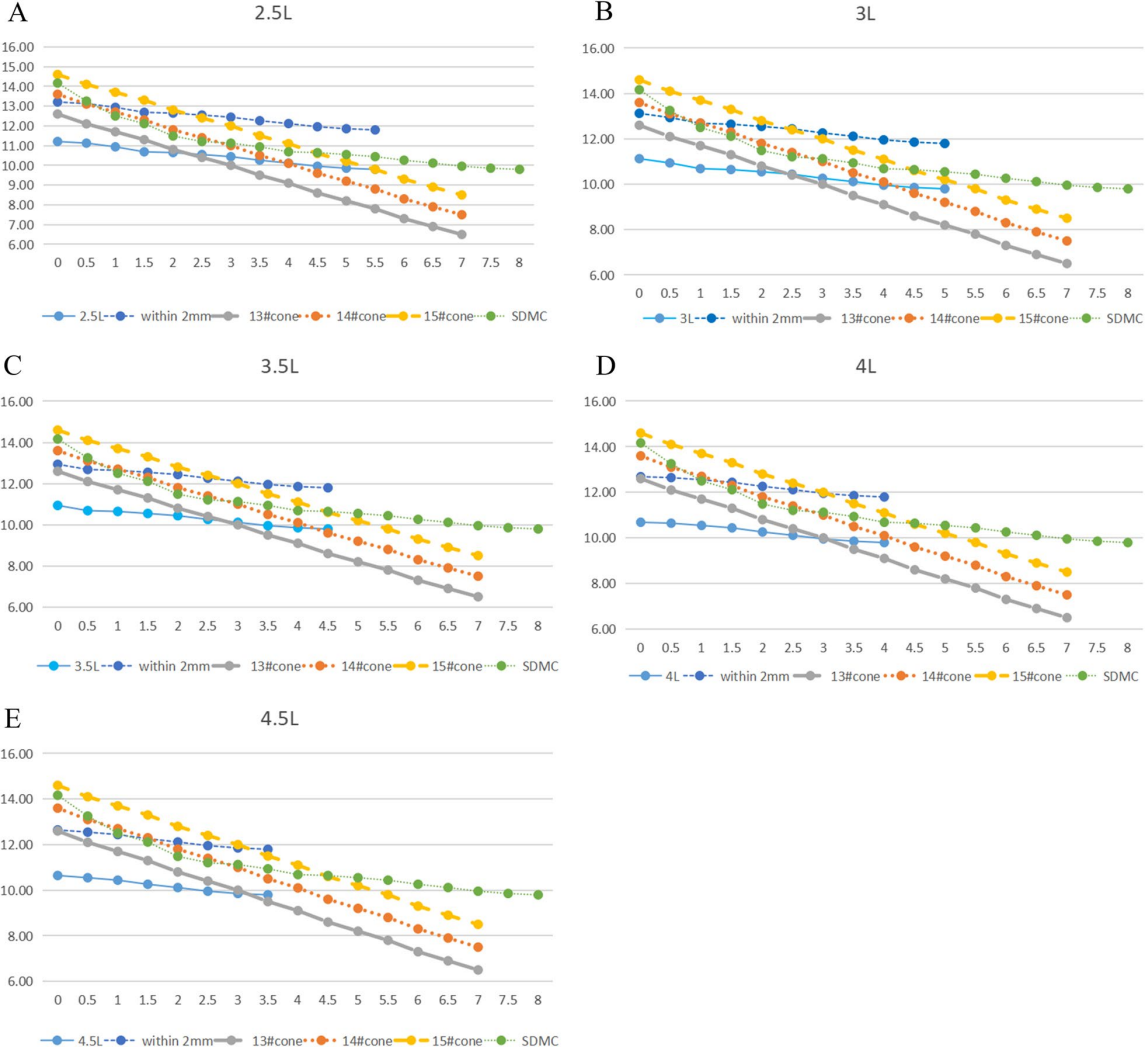
Optimal osteotomy locations for all groups: **A** 2.5L group, **B** 3L group, **C** 3.5L group, **D** 4L group, and **E** 4.5L group. The *x* axis represents the osteotomy location below the LT, the *y* axis represents the diameter (mm)

The optimal locations for osteotomy for all groups were as follows:

1. 2.5L group: just below the LT and 0.5 cm below the LT for the size 13 Wagner cone femoral stem; between 0.5 and 2.5 cm below the LT for the size 14 Wagner cone femoral stem; and between 2.5 and 4 cm below the LT for the size 15 Wagner cone femoral stem (Fig. 4A)
2. 3L group: just below the LT, 0.5 cm below the LT, and 1 cm below the LT for the size 13 Wagner cone femoral stem; between 1 and 2.5 cm below the LT for the size 14 Wagner cone femoral stem; and between 2.5 and 4 cm below the LT for the size 15 Wagner cone femoral stem (Fig. 4B)
3. 3.5L group: just below the LT, 0.5 cm below the LT, and 1 cm below the LT for the size 13 Wagner cone femoral stem; between 1 and 2.5 cm below the LT for the size 14 Wagner cone femoral stem; and between 3 and 4 cm below the LT for the size 15 Wagner cone femoral stem (Fig. 4C)
4. 4L group: between just below the LT and 1.5 cm below the LT for the size 13 Wagner cone femoral stem; between 1.5 and 2.5 cm below the LT for the size 14 Wagner cone femoral stem; and between 3 and 4 cm below the LT for the size 15 Wagner cone femoral stem (Fig. 4D)
5. 4.5L group: between just below the LT and 1.5 cm below the LT for the size 13 Wagner cone femoral stem; between 1.5 and 2.5 cm below the LT for the size 14 Wagner cone femoral stem; and 3.5 cm below the LT for the size 15 Wagner cone femoral stem (Fig. 4E)

### Subtrochanteric osteotomy and the femoral isthmus

For all groups, the level of distal osteotomy should be as follows: (1) 2.5L group, not lower than 5.5 cm below the LT; (2) 3L group, not lower than 5 cm below the LT; (3) 3.5L group, not lower than 4.5 cm below the LT; (4) 4L group, not lower than 4 cm below the LT; and (5) 4.5L group, not lower than 3.5 cm below the LT.

## Discussion

This study was based on the 3D femoral morphology models of 40 patients with Crowe type IV DDH. The parameters of these models (SDMC and bone area) were averaged to simulate subtrochanteric osteotomy at different levels using five osteotomy lengths and different femoral stem sizes. The objective was to (1) evaluate the matching of proximal and distal femoral segments and the femur–femoral stem fitting and (2) analyze the optimal osteotomy location to ensure adequate reduction and stabilization, which may contribute to the bone union at the osteotomy site. The results showed that (1) size 13–15 stems could be implanted well, among which a size 14 femoral stem was more suitable; (2) the optimal osteotomy positions to achieve the minimum EFL were similar when femoral stems of the same size were implanted in five osteotomy lengths; and (3) the optimal osteotomy positions to achieve the minimum EFL were different when femoral stems of different sizes were implanted in five osteotomy lengths. However, the optimal osteotomy position gradually moved distally with an increasing femoral stem size. Non-union of osteotomy may lead to varus angulation, pain, loss of rotational stability, and prosthetic loosening [15]. The incidence of non-union at the osteotomy site ranges from 0 to 7.1% [6, 26, 27]. This study likened subtrochanteric osteotomy to subtrochanteric fracture, for which the three fundamental treatment principles are reduction, stabilization, and rehabilitation, to better understand bone healing after subtrochanteric osteotomy. Osteotomy site matching corresponds to reduction, and the femur–femoral stem fitting corresponds to stabilization. A study has shown that a minimum EFL of 1.5–2.5 cm is sufficient to obtain adequate initial fixation stability with a tapered stem design [22]. Hence, we set 1.5 cm as the minimum EFL and used three indicators to evaluate the reduction and stabilization at the osteotomy site with implanted Wagner cone stems: (1) higher contact area and coincidence rate between the proximal and distal segments; (2) the femoral stem’s EFL at the distal segments is ≥ 1.5 cm; and (3) osteotomy does not involve the isthmus.

Using the contact area and coincidence rate accurately measured with the 3D femoral morphology model to evaluate osteotomy site reduction, we showed that the minimum contact area of the osteotomy site for the five groups was located just below the LT. This result is similar to that of a previous study by Huang et al. [12]. Combined with the SDMC results, this study’s findings suggest the following: an osteotomy site closer to the LT results in a larger femoral medullary cavity diameter and thinner bone cortex; an osteotomy site closer to the femoral isthmus creates a smaller femoral medullary cavity diameter and thicker bone cortex; and an osteotomy site closer to the LT leads to more apparent changes in femoral medullary cavity diameter. Our investigation also revealed that the coincidence rate at the osteotomy site close to the LT (i.e., the level just below the LT to 1 cm below the LT) was significantly lower, indicating that the medullary cavity diameter near the LT was larger than that at or 1.5 cm below the LT. This result is consistent with that of a previous article by Zhang et al. [28]. It was conceivable that the results would lead to obvious step-like changes after splicing the proximal and distal osteotomy segments, and the “reduction” effect of the osteotomy blocks was poor, which may eventually lead to the separation of the proximal and distal osteotomy segments after femoral stem implantation, or may lead to a shorter femoral stem fixation length at the proximal osteotomy segments, affecting the fixation strength (Fig. 2). Therefore, the osteotomy should be performed at or 1.5 cm below the LT. An osteotomy level 1 cm below the LT was selected when the size 13 stem was used to implant the femur with an osteotomy length of 4.5 cm.

EFLs ≥ 1.5 cm were used to evaluate the stabilization at the osteotomy site in this study. The 3D femoral morphology model was measured to accurately describe the femur–Wagner cone femoral stem fitting at each level. Additionally, the SDMC was used to represent the maximum fitting femoral stem diameter at each level of the femoral medullary cavity with different shapes. This study demonstrated that the matching of five osteotomy lengths and three femoral stem sizes can meet the above EFL and that there was a certain rule for the optimal osteotomy level (i.e., the optimal osteotomy level will gradually shift distally with increasing osteotomy lengths, but within a certain range). The details are as follows: (1) when implanting the size 13 Wagner cone femoral stem, the optimal osteotomy level was mainly located at the proximal part of the femoral stem (similar to the levels between just below the LT and 1.5 cm below the LT); (2) when implanting the size 14 Wagner cone femoral stem, the optimal osteotomy level was mainly located at the middle part of the femoral stem (similar to the levels between 0.5 and 2.5 cm below the LT); and (3) when implanting the size 15 Wagner cone femoral stem, the optimal osteotomy level was mainly located at the distal part of the femoral stem (similar to the levels between 2.5 and 4 cm below the LT) (Fig. 4). This rule is consistent with the actual situation because, for a single tapered Wagner cone femoral stem, the proximal part of a size 13 femoral stem is similar to the middle part of a size 14 femoral stem, and the distal part of a size 15 femoral stem, whereas the size of the femur is the same; therefore, the above regular changes occur.

**Fig. 5:**
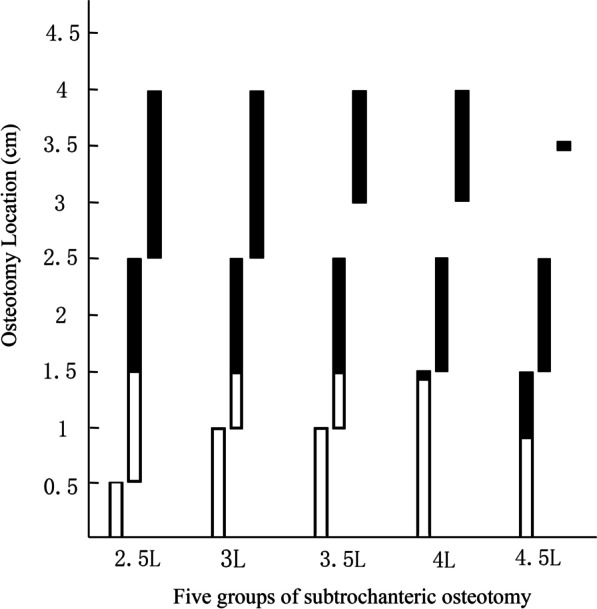
Optimal osteotomy locations under three evaluation indicators for 15 matching modes. The *x* axis represents the subgroups, the *y* axis represents the osteotomy location below the LT. For each group, the left column represents the size 13 stem, the middle column represents the size 14 stem, and the right column represents the size 15 stem. For each group, the height of each column represents the range of optimal osteotomy locations that meet the first and third indicators. The height of the hollow column represents the range of optimal osteotomy locations that do not meet the second indicator. The height of the solid column represents the range of optimal osteotomy locations that simultaneously meet all three indicators

The 15 matching modes (five osteotomy lengths and three Wagner cone femoral stem sizes) were further analyzed, combined with the three evaluation indicators. This study showed that the shortest osteotomy length should not be < 3.5 cm when implanting the size 13 femoral stem because the other matching modes could meet the three evaluation indicators (Fig. 5). Among these matching modes, the range of optimal osteotomy levels for implanting the size 13 stem was the smallest, and the length of the proximal osteotomy segment was short (Fig. 5). There was a risk of poor fixation with the size 13 femoral stem, which may affect bone healing at the osteotomy site. This was associated with the selection of a relatively small femoral stem during the procedure. The range of optimal osteotomy levels for implanting the size 15 stem was greater than that for implanting the size 13 stem (Fig. 5). However, the fixation locations were near the femoral isthmus (Fig. 5), which increased the risk of intraoperative fracture and postoperative thigh pain. This was associated with the selection of a relatively large femoral stem during the procedure. The range of optimal osteotomy levels for implanting the size 14 stem was the largest (Fig. 5), which improved the adjustability of the osteotomy procedure. The optimal osteotomy levels were located at the middle part of the stem, and the press fit was located between the LT and femoral isthmus, which avoided the shortcomings of the above two stems. This was associated with the selection of an appropriate femoral stem during the procedure.

This study introduced the concept of SDMC and compared it with the femoral stem diameter to simulate femoral stem implantation. However, the SDMC was an average value with a standard deviation ranging between 2.2 and 3.6 mm. The size 14 femoral stem was not suitable for all patients with Crowe type IV DDH. There were cases where patients with smaller medullary cavities matched the size 13 femoral stem, and patients with larger medullary cavities matched the size 15 femoral stem. Therefore, matching of the size 14 femoral stem indicated that the above rule applies to the implantation of an appropriately sized femoral stem; i.e., for the five osteotomy lengths (range: 2.5–4.5 cm), the optimal osteotomy level was between 1.5 and 2.5 cm below the LT, and the starting position of the femur–femoral stem alignment was located at the middle part of the femoral stem (4–5 cm below the proximal–medial end of the femoral stem). Hence, during THA combined with subtrochanteric osteotomy for Crowe type IV DDH, the osteotomy length and appropriate femoral stem size should be determined, and the osteotomy plan should coincide with the preoperative plan to improve surgical outcomes. After completion of the osteotomy, femoral stems should be implanted from the smallest to the largest until an appropriate femoral stem size meeting the above conditions is identified.

This study had some limitations. First, the patient population is relatively small and confined to the local region. Previous study has shown that the morphology of the femur (length and curvature radius) varies with differences in population and geography, nutrition and function, and the passage of time [29]. However, due to the low incidence rate of Crowe type IV DDH, as well as variations in ethical requirements and cultural practices across different regions, it is challenging to gather a substantial amount of imaging data for this type of patients on a global scale. Nevertheless, the results of contact area and coincidence rate measurements in this study were similar to those in a previous report [12]. Additionally, through analysis of the 3D femoral morphology model of 40 patients, the results for each patient were accurately shown objectively and representatively. Second, only the subtrochanteric osteotomy lengths of 2.5–4.5 cm were analyzed. However, the data would be extremely large if all osteotomy lengths were analyzed. Additionally, the osteotomy length is usually between 2.5 and 4.5 cm when subtrochanteric osteotomy is performed in clinical practice [7, 18, 19]; therefore, the results are suitable for most situations. Third, the analysis was limited to size 13–15 Wagner cone femoral stems. However, the femoral medullary cavity of patients with Crowe type IV DDH is generally small, and relatively larger femoral stems are rarely used in clinical practice. Furthermore, the results also show that the implantation of a size 15 femoral stem was associated with the selection of a relatively large femoral stem during the procedure. Fourth, Fifth, this study mainly focused on the analysis of imaging data, and the EFL of 1.5 cm was based on the results of previous studies [22]. Thus, finite element analysis and mechanical analysis can be carried out to verify its accuracy in future.

## Conclusions

Subtrochanteric osteotomy at the optimal level can ensure femur–femoral stem fitting and meet the requirements of a higher contact area and coincidence rate to facilitate sufficient reduction and stabilization at the osteotomy site, which may contribute to bone healing. Although the optimal osteotomy level varies with the size of the implanted femoral stem and the length of the subtrochanteric osteotomy, the optimal osteotomy levels for an appropriately sized Wagner cone femoral stem implantation range from 1.5 to 2.5 cm below the LT. This study’s findings may provide a reference for surgeons performing subtrochanteric osteotomy.

## Supplementary Information

**Additional file 1.** Table A1. Height of lesser trochanter for the 40 Crowe type IV DDH hips.

**Additional file 2.** Table A2.1. One-way ANOVA of 2.5L group. Table A2.2. The *q* test of 2.5L group for contact area. Table A2.3. The *q* test of 2.5L group for coincidence rate. The statistical results of contact area and coincidence rate of 2.5L group.

**Additional file 3.** Table A3.1. One-way ANOVA of 3L group. Table A3.2. The *q* test of 3L group for contact area. Table A3.3. The *q* test of 3L group for coincidence rate. The statistical results of contact area and coincidence rate of 3L group.

**Additional file 4.** Table A4.1. One-way ANOVA of 3.5L group. Table A4.2. The *q* test of 3.5L group for contact area. Table A4.3. The *q* test of 3.5L group for coincidence rate. The statistical results of contact area and coincidence rate of 3.5L group.

**Additional file 5.** Table A5.1. One-way ANOVA of 4L group. Table A5.2. The *q* test of 4L group for contact area. Table A5.3. The *q* test of 4L group for coincidence rate. The statistical results of contact area and coincidence rate of 4L group.

**Additional file 6.** Table A6.1. One-way ANOVA of 4.5L group. Table A6.2. The *q* test of 4.5L group for contact area. Table A6.3. The *q* test of 4.5L group for coincidence rate. The statistical results of contact area and coincidence rate of 4.5L group.

## Data Availability

The datasets used and/or analyzed during the current study are available from the corresponding author on reasonable request.

## Abbreviations

DDH: Developmental dysplasia of the hip
LT: Lesser trochanter
THA: Total hip arthroplasty
3D: Three-dimensional
CT: Computed tomography
EFL: Effective fixation length
SDMC: Shortest diameter of the medullary cavity
ANOVA: Analysis of variance
