# Supplementary material for: Three-dimensional femur morphology analysis for the optimal location of subtrochanteric osteotomy with an implanted Wagner cone stem in total hip arthroplasty for Crowe type IV developmental dysplasia of the hip
Source: J Orthop Surg Res. 2023 Jun 5;18:410. doi: 10.1186/s13018-023-03901-7 (PMC10243028; doi:10.1186/s13018-023-03901-7)
Supplement: Supplementary file 1 — Additional file 1. Table A1. Height of lesser trochanter for the 40 Crowe type IV DDH hips. [file 13018_2023_3901_MOESM1_ESM.docx]

**Additional file 1**

Table A1. Height of lesser trochanter for the 40 Crowe type IV DDH hips

| Cases | Height of lesser trochanter |
| --- | --- |
|  |  |
| 1 | 36 |
| 2 | 34.5 |
| 3 | 20.4 |
| 4 | 24.8 |
| 5 | 24.3 |
| 6 | 21.2 |
| 7 | 18.7 |
| 8 | 26.5 |
| 9 | 22.3 |
| 10 | 27.7 |
| 11 | 21.1 |
| 12 | 23.5 |
| 13 | 28.7 |
| 14 | 34.3 |
| 15 | 19.8 |
| 16 | 28.2 |
| 17 | 24.9 |
| 18 | 22.5 |
| 19 | 23.8 |
| 20 | 21.2 |

| Cases | Height of lesser trochanter |
| --- | --- |
|  |  |
| 21 | 21.3 |
| 22 | 28 |
| 23 | 28.6 |
| 24 | 18.6 |
| 25 | 33.7 |
| 26 | 23.2 |
| 27 | 25 |
| 28 | 19.8 |
| 29 | 28.9 |
| 30 | 28.4 |
| 31 | 32 |
| 32 | 23.6 |
| 33 | 24.1 |
| 34 | 23.6 |
| 35 | 23.5 |
| 36 | 23.4 |
| 37 | 34.9 |
| 38 | 39.5 |
| 39 | 23.8 |
| 40 | 28.5 |

The average height of lesser trochanter is 25.9 mm.
