# Supplementary material for: Three-dimensional femur morphology analysis for the optimal location of subtrochanteric osteotomy with an implanted Wagner cone stem in total hip arthroplasty for Crowe type IV developmental dysplasia of the hip
Source: J Orthop Surg Res. 2023 Jun 5;18:410. doi: 10.1186/s13018-023-03901-7 (PMC10243028; doi:10.1186/s13018-023-03901-7)
Supplement: Supplementary file 2 — Additional file 2. Table A2.1. One-way ANOVA of 2.5L group. Table A2.2. The q test of 2.5L group for contact area. Table A2.3. The q test of 2.5L group for coincidence rate. The statistical results of contact area and coincidence rate of 2.5L group. [file 13018_2023_3901_MOESM2_ESM.doc]

|  | | Sum of Squares | df. | Mean Squares | F | Sig. |
| --- | --- | --- | --- | --- | --- | --- |
| Contact Area_2.5L | Inter-group | 270403.462 | 11 | 24582.133 | 7.151 | .000 |
|  | Intra-group | 1608882.788 | 468 | 3437.784 |  |  |
|  | Total | 1879286.249 | 479 |  |  |  |
| Coincidence Rate_2.5L | Inter-group | 2.701 | 11 | .246 | 30.760 | .000 |
|  | Intra-group | 3.736 | 468 | .008 |  |  |
|  | Total | 6.437 | 479 |  |  |  |

**Additional file 2**

Table A2.1. One-way ANOVA of 2.5L group

Table A2.2. The q-test of 2.5L group for contact area

| Level (cm) | N | Subset for Alpha = 0.05 | |  |
| --- | --- | --- | --- | --- |
|  |  | 1 | 2 |  |
| 0 | 40 | 202.2883 |  |  |
| 0.5 | 40 |  | 229.5178 |  |
| 1 | 40 |  | 252.8923 | 252.8923 |
| 1.5 | 40 |  |  | 261.8610 |
| 2 | 40 |  |  | 269.4983 |
| 2.5 | 40 |  |  | 273.1687 |
| 5.5 | 40 |  |  | 273.5831 |
| 5 | 40 |  |  | 279.0537 |
| 3 | 40 |  |  | 279.5471 |
| 3.5 | 40 |  |  | 280.0378 |
| 4 | 40 |  |  | 281.7060 |
| 4.5 | 40 |  |  | 283.7410 |
| Sig. |  | 1.000 | .075 | .357 |

Table A2.3. The q-test of 2.5L group for coincidence rate

| Level (cm) | N | Subset for Alpha = 0.05 | | | |  |
| --- | --- | --- | --- | --- | --- | --- |
|  |  | 1 | 2 | 3 | 4 | |
| 0 | 40 | .62745 |  |  |  | |
| 0.5 | 40 |  | .71842 |  |  | |
| 1 | 40 |  |  | .79662 |  | |
| 1.5 | 40 |  |  | .82246 | .82246 | |
| 2 | 40 |  |  |  | .84508 | |
| 2.5 | 40 |  |  |  | .85601 | |
| 5.5 | 40 |  |  |  | .86336 | |
| 3 | 40 |  |  |  | .86668 | |
| 3.5 | 40 |  |  |  | .87661 | |
| 5 | 40 |  |  |  | .87729 | |
| 4 | 40 |  |  |  | .88055 | |
| 4.5 | 40 |  |  |  | .88227 | |
| Sig. |  | 1.000 | 1.000 | .196 | .071 | |
