# Supplementary material for: Three-dimensional femur morphology analysis for the optimal location of subtrochanteric osteotomy with an implanted Wagner cone stem in total hip arthroplasty for Crowe type IV developmental dysplasia of the hip
Source: J Orthop Surg Res. 2023 Jun 5;18:410. doi: 10.1186/s13018-023-03901-7 (PMC10243028; doi:10.1186/s13018-023-03901-7)
Supplement: Supplementary file 3 — Additional file 3. Table A3.1. One-way ANOVA of 3L group. Table A3.2. The q test of 3L group for contact area. Table A3.3. The q test of 3L group for coincidence rate. The statistical results of contact area and coincidence rate of 3L group. [file 13018_2023_3901_MOESM3_ESM.doc]

|  | | Sum of Squares | df. | Mean Squares | F | Sig. |
| --- | --- | --- | --- | --- | --- | --- |
| Contact Area_3L | Inter-group | 246756.700 | 10 | 24675.670 | 7.282 | .000 |
|  | Intra-group | 1453724.388 | 429 | 3388.635 |  |  |
|  | Total | 1700481.089 | 439 |  |  |  |
| Coincidence Rate_3L | Inter-group | 2.465 | 10 | .246 | 26.253 | .000 |
|  | Intra-group | 4.027 | 429 | .009 |  |  |
|  | Total | 6.492 | 439 |  |  |  |

**Additional file 3**

Table A3.1. One-way ANOVA of 3L group

Table A3.2. The q-test of 3L group for contact area

| Level (cm) | N | Subset for Alpha = 0.05 | |  |
| --- | --- | --- | --- | --- |
|  |  | 1 | 2 |  |
| 0 | 40 | 197.5262 |  |  |
| 0.5 | 40 |  | 225.9994 |  |
| 1 | 40 |  | 248.7599 | 248.7599 |
| 1.5 | 40 |  |  | 257.6151 |
| 2 | 40 |  |  | 265.3242 |
| 5 | 40 |  |  | 266.0617 |
| 2.5 | 40 |  |  | 270.7979 |
| 3 | 40 |  |  | 271.8927 |
| 4.5 | 40 |  |  | 273.6741 |
| 3.5 | 40 |  |  | 275.0904 |
| 4 | 40 |  |  | 277.8513 |
| Sig. |  | 1.000 | .081 | .385 |

Table A3.3. The q-test of 3L group for coincidence rate

| Level (cm) | N | Subset for Alpha = 0.05 | | | | |
| --- | --- | --- | --- | --- | --- | --- |
|  |  | 1 | 2 | 3 | 4 |  |
| 0 | 40 | .61342 |  |  |  |  |
| 0.5 | 40 |  | .70908 |  |  |  |
| 1 | 40 |  |  | .78089 |  |  |
| 1.5 | 40 |  |  | .80632 | .80632 |  |
| 2 | 40 |  |  | .83059 | .83059 |  |
| 5 | 40 |  |  |  | .83963 |  |
| 2.5 | 40 |  |  |  | .84073 |  |
| 3 | 40 |  |  |  | .85193 |  |
| 3.5 | 40 |  |  |  | .86017 |  |
| 4.5 | 40 |  |  |  | .86171 |  |
| 4 | 40 |  |  |  | .86445 |  |
| Sig. |  | 1.000 | 1.000 | .058 | .131 |  |
