# Supplementary material for: Three-dimensional femur morphology analysis for the optimal location of subtrochanteric osteotomy with an implanted Wagner cone stem in total hip arthroplasty for Crowe type IV developmental dysplasia of the hip
Source: J Orthop Surg Res. 2023 Jun 5;18:410. doi: 10.1186/s13018-023-03901-7 (PMC10243028; doi:10.1186/s13018-023-03901-7)
Supplement: Supplementary file 5 — Additional file 5. Table A5.1. One-way ANOVA of 4L group. Table A5.2. The q test of 4L group for contact area. Table A5.3. The q test of 4L group for coincidence rate. The statistical results of contact area and coincidence rate of 4L group. [file 13018_2023_3901_MOESM5_ESM.doc]

|  | | Sum of Squares | df. | Mean Squares | F | Sig. |
| --- | --- | --- | --- | --- | --- | --- |
| Contact Area_4L | Inter-group | 196530.102 | 8 | 24566.263 | 7.211 | .000 |
|  | Intra-group | 1195835.250 | 351 | 3406.938 |  |  |
|  | Total | 1392365.352 | 359 |  |  |  |
| Coincidence Rate_4L | Inter-group | 2.024 | 8 | .253 | 23.110 | .000 |
|  | Intra-group | 3.842 | 351 | .011 |  |  |
|  | Total | 5.866 | 359 |  |  |  |

**Additional file 5**

Table A5.1. One-way ANOVA of 4L group

Table A5.2. The q-test of 4L group for contact area

| Level (cm) | N | Subset for Alpha = 0.05 | |  |
| --- | --- | --- | --- | --- |
|  |  | 1 | 2 |  |
| 0 | 40 | 191.5179 |  |  |
| 0.5 | 40 |  | 218.3811 |  |
| 1 | 40 |  | 240.5999 | 240.5999 |
| 1.5 | 40 |  |  | 250.4024 |
| 2 | 40 |  |  | 257.5257 |
| 4 | 40 |  |  | 257.6914 |
| 3.5 | 40 |  |  | 260.4195 |
| 2.5 | 40 |  |  | 262.8847 |
| 3 | 40 |  |  | 265.4992 |
| Sig. |  | 1.000 | .090 | .477 |

Table A5.3. The q-test of 4L group for coincidence rate

| Level (cm) | N | Subset for Alpha = 0.05 | | | | |
| --- | --- | --- | --- | --- | --- | --- |
|  |  | 1 | 2 | 3 | 4 |  |
| 0 | 40 | .59458 |  |  |  |  |
| 0.5 | 40 |  | .68016 |  |  |  |
| 1 | 40 |  |  | .75086 |  |  |
| 1.5 | 40 |  |  | .77415 | .77415 |  |
| 2 | 40 |  |  |  | .80617 |  |
| 4 | 40 |  |  |  | .81307 |  |
| 3.5 | 40 |  |  |  | .81964 |  |
| 2.5 | 40 |  |  |  | .82300 |  |
| 3 | 40 |  |  |  | .82594 |  |
| Sig. |  | 1.000 | 1.000 | .320 | .234 |  |
